# Clinical value of cholinesterase in patients treated with radical nephroureterectomy for upper urinary tract carcinoma

**DOI:** 10.1007/s00345-023-04449-1

**Published:** 2023-06-09

**Authors:** Markus von Deimling, David D’Andrea, Benjamin Pradere, Ekaterina Laukhtina, Takafumi Yanagisawa, Tatsushi Kawada, Muhammad Majdoub, Pawel Rajwa, Maximilian Pallauf, Nirmish Singla, Francesco Soria, Vitaly Margulis, Piotr Chlosta, Pierre I. Karakiewicz, Morgan Roupret, Jeremy Yuen-Chun Teoh, Margit Fisch, Michael Rink, Marco Moschini, Yair Lotan, Shahrokh F. Shariat

**Affiliations:** 1 Department of Urology, Comprehensive Cancer Center, Vienna General Hospital, Medical University of Vienna, Währinger Gürtel 18-20, 1090 Vienna, Austria; 2 Department of Urology, University Medical Center Hamburg-Eppendorf, Hamburg, Germany; 3 Department of Urology, La Croix Du Sud Hospital, Quint-Fonsegrives, France; 4 Institute for Urology and Reproductive Health, Sechenov University, Moscow, Russia; 5 Department of Urology, The Jikei University School of Medicine, Tokyo, Japan; 6 Department of Urology, Okayama University Graduate School of Medicine, Dentistry and Pharmaceutical Sciences, Okayama, Japan; 7 Department of Urology, Hillel Yaffe Medical Center, Hadera, Israel; 8 Department of Urology, Medical University of Silesia, Zabrze, Poland; 9 Departments of Urology and Oncology, The James Buchanan Brady Urological Institute, Johns Hopkins University School of Medicine, Baltimore, MD USA; 10 Department of Urology, University Hospital Salzburg, Paracelsus Medical University, Salzburg, Austria; 11 Division of Urology, Department of Surgical Sciences, San Giovanni Battista Hospital, University of Studies of Torino, Turin, Italy; 12 Department of Urology, University of Texas Southwestern Medical Center, Dallas, TX USA; 13 Department of Urology, Jagiellonian University, Cracow, Poland; 14 Cancer Prognostics and Health Outcomes Unit, Division of Urology, University of Montreal Health Center, Montreal, Canada; 15 Sorbonne University, GRC 5 Predictive Onco-Uro, AP-HP, Urology, Pitie-Salpetriere Hospital, Paris, France; 16 S.H. Ho Urology Centre, Department of Surgery, Prince of Wales Hospital, The Chinese University of Hong Kong, Hong Kong, China; 17 Department of Urology, Urological Research Institute, Vita-Salute University, San Raffaele Scientific Institute, Milan, Italy; 18 Karl Landsteiner Institute of Urology and Andrology, Vienna, Austria; 19 Department of Urology, Weill Cornell Medical College, New York, NY USA; 20 Department of Urology, Second Faculty of Medicine, Charles University, Prague, Czech Republic

**Keywords:** Urothelial neoplasm, Upper urinary tract urothelial carcinoma, Biomarker, Cholinesterase, Decision curve analysis

## Abstract

**Purpose:**

To evaluate the prognostic value and the clinical impact of preoperative serum cholinesterase (ChoE) levels on decision-making in patients treated with radical nephroureterectomy (RNU) for clinically non-metastatic upper tract urothelial cancer (UTUC).

**Methods:**

A retrospective review of an established multi-institutional UTUC database was performed. We evaluated preoperative ChoE as a continuous and dichotomized variable using a visual assessment of the functional form of the association of ChoE with cancer-specific survival (CSS). We used univariable and multivariable Cox regression models to establish its association with recurrence-free survival (RFS), CSS, and overall survival (OS). Discrimination was evaluated using Harrell’s concordance index. Decision curve analysis (DCA) was used to assess the impact on clinical decision-making of preoperative ChoE.

**Results:**

A total of 748 patients were available for analysis. Within a median follow-up of 34 months (IQR 15–64), 191 patients experienced disease recurrence, and 257 died, with 165 dying of UTUC. The optimal ChoE cutoff identified was 5.8 U/l. ChoE as continuous variable was significantly associated with RFS (*p* < 0.001), OS (*p* < 0.001), and CSS (*p* < 0.001) on univariable and multivariable analyses. The concordance index improved by 8%, 4.4%, and 7% for RFS, OS, and CSS, respectively. On DCA, including ChoE did not improve the net benefit of standard prognostic models.

**Conclusion:**

Despite its independent association with RFS, OS, and CSS, preoperative serum ChoE has no impact on clinical decision-making. In future studies, ChoE should be investigated as part of the tumor microenvironment and assessed as part of predictive and prognostic models, specifically in the setting of immune checkpoint-inhibitor therapy.

**Supplementary Information:**

The online version contains supplementary material available at 10.1007/s00345-023-04449-1.

## Introduction

Radical nephroureterectomy (RNU) with bladder cuff resection is the gold standard treatment for high-risk non-metastatic upper tract urothelial cancer (UTUC) in patients with adequate renal function [1]. Approximately 30% of patients with UTUC experience disease recurrence, and long-term survival after recurrence is poor, emphasizing the urgent need for accurate outcome prediction and biomarker development [2–4]. Pretreatment inflammatory blood-based biomarkers received increasing attention in risk stratification strategies, as they are often easy to assess and inexpensive. However, due to a lack of data, external validation, and limitations related to data quality and study design, they are often not implemented in the clinical setting [5, 6].

Abnormal serum levels of cholinesterase (ChoE, also butyrylcholinesterase or pseudocholinesterase), a hydrolytic enzyme synthesized in the liver, have been associated with different pathologies, including malnutrition, non-liver diseases, chronic inflammation, and cancer [7–10]. Lower pretreatment levels of ChoE were recently associated with worse survival outcomes in several urologic cancers, including prostate cancer, non-muscle-invasive, and muscle-invasive bladder cancer [11–14]. It has been suggested that decreased ChoE levels may be an expression of hepatic dysfunction due to tumor cachexia and the underlying systemic disease. Indeed, gene expression of ChoE is altered in several tumor entities [15, 16]. While ChoE is involved in cellular proliferation and differentiation, the exact molecular interplay ChoE with tumor cells remains elusive [17].

In addition, two small studies assessed the role of pretreatment ChoE levels in patients with UTUC [18, 19], but neither thoroughly evaluated the association of pretreatment ChoE levels with known adverse pathological features associated with worse survival outcomes, such as tumor necrosis and sessile tumor architecture [20, 21]. Moreover, both studies were limited by their standard statistical approach, the small sample size, and single-center study design [6, 18, 19].

Against this backdrop, we aimed to evaluate the prognostic value and the impact on clinical decision-making of preoperative serum levels of ChoE in a large, multicenter cohort of patients treated with RNU for clinically non-metastatic UTUC. We hypothesized that low preoperative serum levels of ChoE would predict survival outcomes.

## Materials and methods

### Patient selection

This was a multicenter, institutional review board-approved (IRB No. 1566/2017) study. We retrospectively queried our established UTUC database to identify all patients who underwent RNU for clinically non-metastatic UTUC (cTany N0 M0) between 1990 and 2008 with preoperative measurement of serum ChoE levels within 30 days before RNU. We excluded all metastatic patients as well as patients with missing preoperative serum ChoE level, clinical and/or pathological staging, or survival outcomes.

Serum ChoE level was routinely measured during preoperative evaluation to assess liver function. Serum samples were analyzed according to the manufacturer’s instructions at each institution. Clinical preoperative staging consisted of urinary cytology, endoscopic evaluation of the bladder as well as routine imaging, including computerized tomography or magnetic resonance imaging of the pelvis, abdomen, and thorax.

All patients underwent open or laparoscopic RNU with or without lymph node dissection at the surgeon’s discretion based on guideline recommendations at the time of RNU. All tumor specimens were staged according to the most recent American Joint Committee on Cancer (AJCC) Cancer Staging Manual’s TNM classification. Experienced, dedicated uropathologists at each center performed histological examinations. There was no centralized pathology.

The primary endpoints consisted of recurrence-free (RFS), cancer-specific (CSS), and overall survival (OS). Risk-adapted follow-up was performed according to contemporary guideline recommendations and included clinical and laboratory follow-up, urinary cytology, endoscopic bladder surveillance, and regular cross-sectional imaging, as appropriate. Time-to-event variables were calculated from the date of RNU to either tumor recurrence, death of UTUC, or death from any cause. Cause of death was determined by the treating physician, medical chart review, or death certificates [22]. Tumor recurrence was defined as the occurrence of locoregional recurrence(s) or distant metastasis on radiological imaging. Postoperative tumor manifestation in the bladder was not considered a recurrence. Patients were censored at their last follow-up.

### Calculation of ChoE cutoff

To determine the optimal cutoff value of preoperative serum ChoE, we visually assessed the functional form of the association of preoperative serum ChoE levels with CSS. A multivariable Cox proportional hazards model was fitted, including continuously coded preoperative ChoE levels. We then plotted the Martingale residuals of the underlying Cox proportional hazards model against the ChoE levels. Martingale residuals display the difference between the observed and the expected (based on the Cox model) number of events (CSS). While patients with residuals > 0 are at increased risk for CSS, the optimal cutoff is identified at the x-intersection of the smoothed Martingale plot.

### Statistical analysis

Our statistical analysis followed several steps. First, patients were stratified by dichotomized preoperative ChoE levels to assess baseline, treatment, and histopathological characteristics. We report categorical variables as frequencies and proportions and continuously coded variables with medians and interquartile ranges (IQR). To test for normal distribution, we employed the Shapiro–Wilk test for normality. We compared continuously coded variables with non-normal distribution using the Wilcoxon rank sum test and those with normal distribution using a two-sample independent *t* test. We compared categorical variables using the Chi-square or Fisher’s exact test, as appropriate.

Second, we used the Kaplan–Meier method and log-rank tests to compare survival between groups. Univariable and multivariable Cox regression models were fitted and adjusted for the effects of pathological tumor and node stage, tumor grade, presence of concomitant carcinoma in situ, adverse pathological features such as lymphovascular invasion or sessile tumor architecture, and receipt of perioperative chemotherapy to test the association of preoperative ChoE levels (continuously coded) with RFS, OS, and CSS. The discriminative power of the multivariable models and the additional prognostic value of ChoE was assessed using Harrell’s concordance index (C-index). Similarly, to assess the impact of non-cancer-specific mortality on survival outcomes, we fitted univariable and multivariable competing risks models according to Fine and Gray predicting cancer-specific mortality (CSM) with and without continuously coded preoperative ChoE levels. Lastly, we evaluated the clinical benefit of the predictive models using decision curve analysis (DCA) [23].

Statistical analysis was performed using R version 4.1.2 (R Foundation for Statistical Computing, Vienna, Austria). All tests were two sided, and *p* values < 0.05 were considered statistically significant.

## Results

### Baseline characteristics and association of preoperative serum ChoE with clinicopathologic features

A total of 748 patients were available for analysis. The optimal cutoff for preoperative serum ChoE was 5.8 U/l (Supplementary Fig. 1). According to this cutoff, 259 and 489 patients had low and high ChoE values, respectively (Table 1). When stratified by this ChoE cutoff, a higher proportion of patients with low ChoE value received adjuvant chemotherapy (*p* < 0.01) or external beam radiation (*p* = 0.01) compared to patients with high ChoE values. Moreover, patients with ChoE ≤ 5.8 U/l had higher rates of adverse pathological features, including advanced pathological tumor stage (*p* < 0.01), high-grade or multifocal tumors (*p* < 0.01), lymphovascular invasion (*p* < 0.01), and sessile tumor architecture (*p* < 0.01) (Table 1).

**Table 1:** Clinicopathologic features of 748 patients treated with radical nephroureterectomy (RNU) for clinically non-metastatic upper tract urothelial cancer stratified by preoperative serum cholinesterase (ChoE) level

	Overall	High	Low	*p* value
*n*	748	489	259 (34.6)	
Age, median (IQR)	69.6 (62.5, 76)	69 (62.4, 76)	70.3 (63, 76.7)	0.42
Female gender, *n* (%)	328 (43.9)	208 (42.5)	120 (46.3)	0.36
Left side, *n* (%)	402 (53.7)	257 (52.6)	145 (56.0)	0.41
Laparoscopic RNU, *n* (%)	164 (21.9)	119 (24.3)	45 (17.4)	**0.04**
Lymphadenectomy performed, *n* (%)	181 (24.2)	111 (22.7)	70 (27.0)	0.22
Nodes removed, median (IQR)	4 (2–7)	4 (2–7)	4 (1–7)	0.8
pT stage, *n* (%)				** < 0.01**
Ta/Tis	135 (18.0)	108 (22.1)	27 (10.4)	
T1	218 (29.1)	165 (33.7)	53 (20.5)	
T2	131 (17.5)	80 (16.4)	51 (19.7)	
T3	220 (29.4)	118 (24.1)	102 (39.4)	
T4	44 (5.9)	18 (3.7)	26 (10.0)	
High grade, *n* (%)	563 (75.3)	339 (69.3)	224 (86.5)	** < 0.01**
pN stage, *n* (%)				**0.01**
N0	119 (15.9)	81 (16.6)	38 (14.7)	
N1	62 (8.3)	30 (6.1)	32 (12.4)	
Nx	567 (75.8)	378 (77.3)	189 (73.0)	
LVI, *n* (%)	163 (21.8)	88 (18.0)	75 (29.0)	** < 0.01**
Sessile architecture, *n* (%)	142 (19.0)	75 (15.3)	67 (25.9)	** < 0.01**
Concomitant CIS, *n* (%)	151 (20.2)	93 (19.0)	58 (22.4)	0.32
Necrosis, *n* (%)	104 (13.9)	70 (14.3)	34 (13.1)	0.74
Multifocal, *n* (%)	160 (21.4)	87 (17.8)	73 (28.2)	** < 0.01**
Variant histology, *n* (%)	85 (11.4)	57 (11.7)	28 (10.8)	0.82
Location, *n* (%)				0.12
Renal pelvis	526 (70.3)	341 (69.7)	185 (71.4)	
Ureter	206 (27.5)	141 (28.8)	65 (25.1)	
Both	16 (2.1)	7 (1.4)	9 (3.5)	
Neo-adjuvant chemotherapy, *n* (%)	19 (2.5)	9 (1.8)	10 (3.9)	0.15
Adjuvant chemotherapy, *n* (%)	74 (9.9)	35 (7.2)	39 (15.1)	** < 0.01**
Adjuvant external beam radiation, *n* (%)	18 (2.4)	6 (1.2)	12 (4.6)	**0.01**
ChoE, median (IQR)	6.47 (4.73, 7.30)	7.01 (6.49, 7.89)	4.04 (3.27, 4.85)	** < 0.01**
*CIS* carcinoma in situ, *IQR* interquartile range, *LVI* lymphovascular invasion				

*p* value < 0.05

### Association of ChoE levels with survival outcomes

**Fig. 1:**
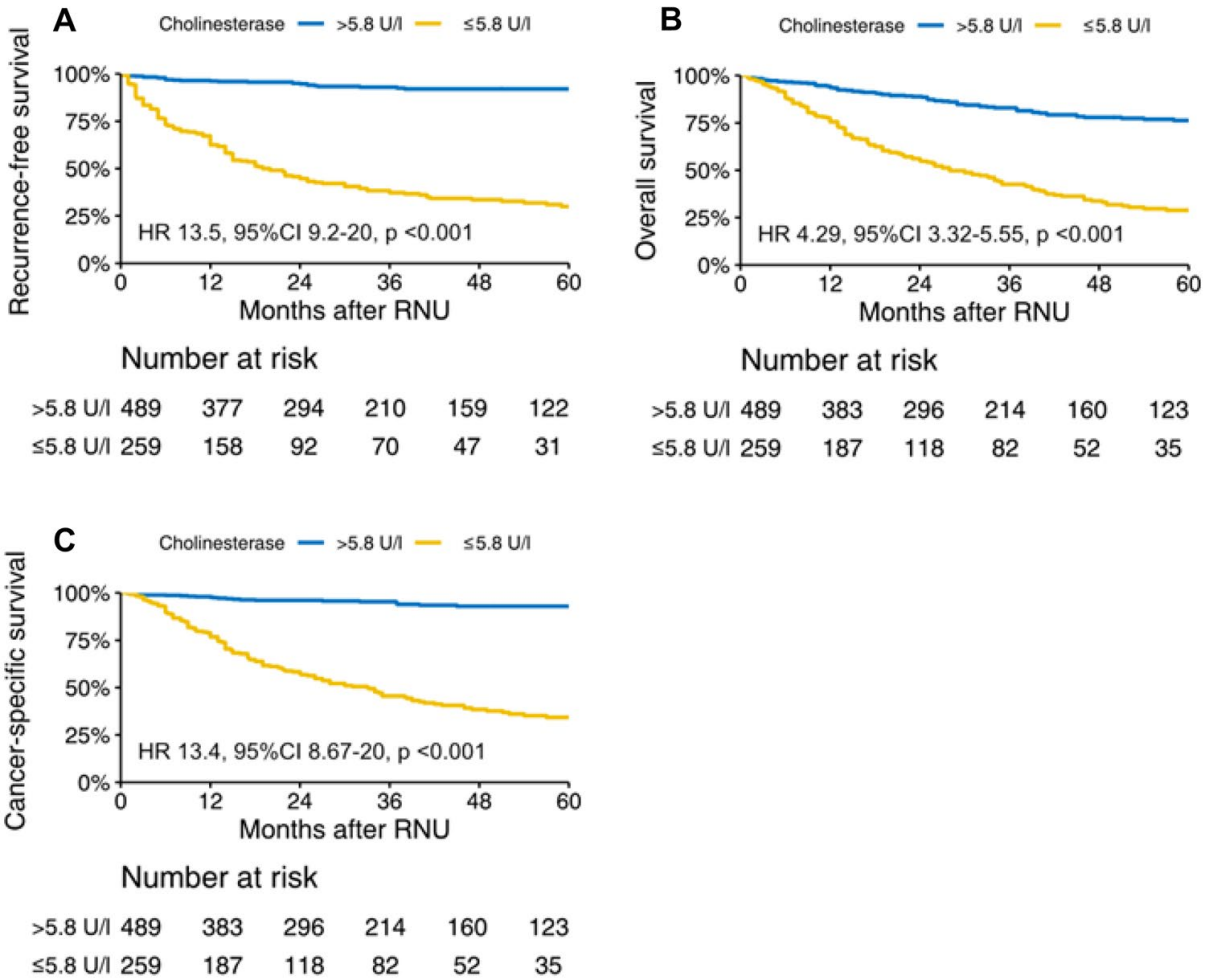
Kaplan–Meier curves for (**A**) recurrence-free, **B** overall, and **C** cancer-specific survival for 748 patients treated with radical nephroureterectomy (RNU) for clinically non-metastatic upper urinary tract urothelial carcinoma stratified by preoperative serum cholinesterase levels. *CI* confidence interval, *HR* hazard ratio

With a median follow-up of 34 months (IQR 15–64), 191 patients (26%) experienced disease recurrence, 257 (34%) died of any cause, and 165 (22%) died of UTUC. A low ChoE level was significantly associated with worse RFS, OS, and CSS (*p* < 0.001) (Fig. 1). This was confirmed for CSM on competing risks analysis (Supplementary Fig. 2). Five-year RFS, OS, and CSS rates were 30% (95% CI 0.34–0.37), 28.9% (95% CI 0.23–0.36), and 34.3% (95% CI 0.28–0.42) for low ChoE values and 92% (95% CI 0.89–0.95), 76.3% (95% CI 71.6–81.3), and 92.9% (95% CI 0.9–0.96) for high ChoE values, respectively.

On univariable Cox regression analyses, ChoE, evaluated as a continuous variable, was significantly associated with shorter RFS (HR 0.53 95% CI 0.49–0.58 *p* < 0.001), OS (HR 0.68 95% CI 0.64–0.73 *p* < 0.001), and CSS (HR 0.54 95% CI 0.49–0.59 *p* < 0.001). When adjusting for the effect of established clinical and histopathological confounders, ChoE remained independently associated with survival outcomes (all *p* < 0.001; Table 2). The addition of ChoE improved the discrimination of standard predictive models by 8%, 4.4%, and 7% for RFS, OS, and CSS, respectively (Table 2). The results remained similar on multivariable analyses when accounting for competing risks. Yet, high-grade disease was no longer associated with CSM (Supplementary Table 1).

**Table 2:** Multivariable Cox regression analysis assessing the association of clinicopathologic features with recurrence-free survival (RFS), overall survival (OS), and cancer-specific survival (CSS) and the accuracy of the models after implementation of cholinesterase (ChoE) in 748 patients treated with radical nephroureterectomy for clinically non-metastatic upper tract urothelial cancer

	RFS			OS			CSS		
	HR	95%CI	*p* value	HR	95%CI	*p* value	HR	95%CI	*p* value
Stage (ref Ta/Tis)									
T1	1.93	0.82–4.53	0.1	0.93	0.56–1.57	0.8	1.59	0.63–4.02	0.33
T2	4.04	1.74–9.37	**0.001**	1.37	0.8–2.33	0.2	3.59	1.45–8.89	**0.005**
T3	6.93	3.05–15.7	** < 0.001**	2.51	1.52–4.14	** < 0.001**	6.11	2.53–14.7	** < 0.001**
T4	31.3	12.8–76.1	** < 0.001**	9.66	5.24–17.8	** < 0.001**	26.2	10.1–68.3	** < 0.001**
High grade	1.48	0.89–2.45	0.13	1.81	1.2–2.72	**0.004**	1.82	1.03–3.19	**0.04**
Concomitant CIS	1.29	0.92–1.82	0.13	1.12	0.82–1.54	0.4	0.97	0.66–1.44	0.89
N stage (ref pN0)									
pN1	1.51	0.92–2.49	0.1	1.62	0.96–2.7	0.07	1.55	0.88–2.74	0.13
pNx	0.83	0.56–1.25	0.38	1.14	0.78–1.67	0.5	0.89	0.57–1.41	0.64
Adverse pathologic features*	1.11	0.79–1.56	0.53	1.42	1.06–1.9	**0.01**	1.38	0.96–1.98	0.08
Perioperative chemotherapy	1.76	1.21–2.55	**0.003**	0.83	0.56–1.23	0.3	1.13	0.73–1.74	0.58
	C-index 0.803			C-index 0.743			C-index 0.807		
ChoE (continuously coded)	0.58	0.53–0.63	** < 0.001**	0.73	0.68–0.78	** < 0.001**	0.59	0.54–0.65	** < 0.001**
	C-index 0.883			C-index 0.787			C-index 0.877		

*CI* confidence interval, *HR* hazard ratio

*One or multiple features including lymphovascular invasion, tumor necrosis, tumor architecture and variant histology

*p* value < 0.05

### Decision curve analysis

**Fig. 2:**
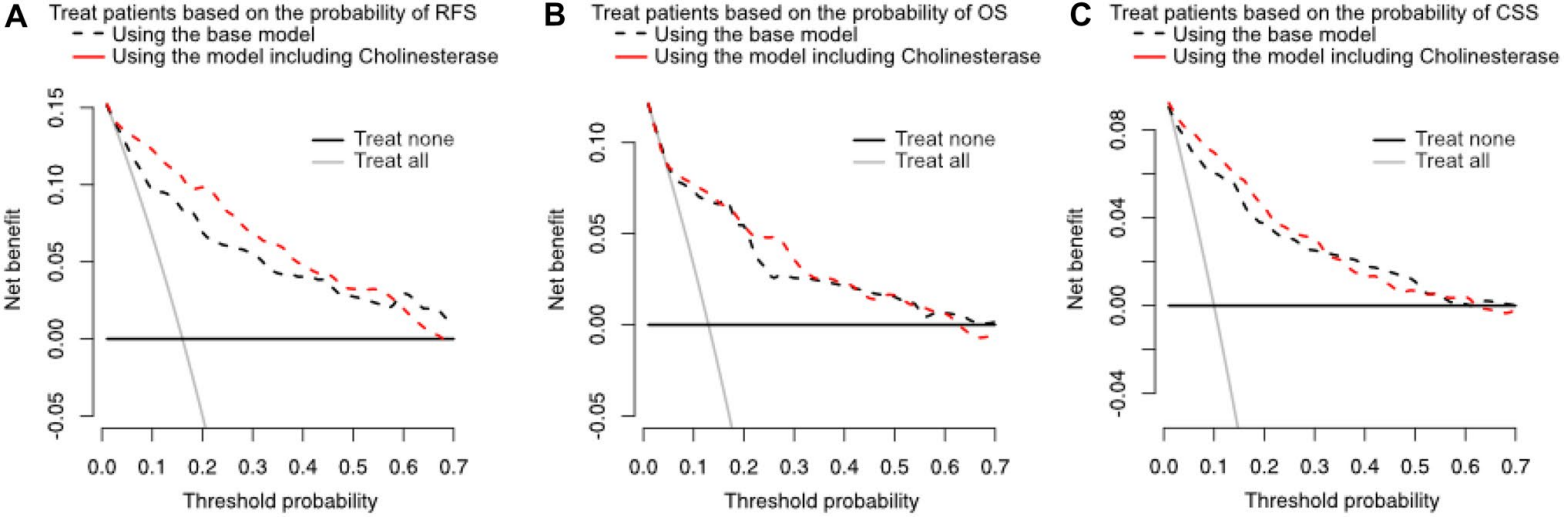
Decision curve analysis for the additional net benefit provided by preoperative serum levels of cholinesterase added to a base model for prediction of (**A**) recurrence-free (RFS), **B** overall (OS), and **C** cancer-specific survival (CSS) in 748 patients treated with radical nephroureterectomy (RNU) for clinically non-metastatic upper urinary tract urothelial carcinoma

On DCA, the addition of preoperative serum ChoE to the base models did not improve their clinical net benefit for the prediction of RFS, OS, or CSS by a significant margin (Fig. 2).

## Discussion

In this study, we assessed the role of preoperative serum ChoE levels in patients treated with RNU for clinically non-metastatic UTUC in a large multicenter cohort. We found a statistically significant association between preoperative serum ChoE levels and RFS, OS, and CSS. In addition, applying a cutoff of 5.8 U/l, a low ChoE value correlated with the presence of adverse clinicopathological features, including higher pathological tumor stage, more high-grade and multifocal tumors, lymphovascular invasion, and sessile tumor architecture. For descriptive statistics and survival analysis, dichotomized ChoE levels were used, whereas on univariable and multivariable Cox models, ChoE was continuously coded. These findings are in agreement with those of previous studies on the role of ChoE in genitourinary malignancies [11–13, 15, 18, 19].

Current risk stratification for UTUC is based on clinical and pathologic features [1]. However, these models suffer from moderate discrimination and a lack of external validation [24]. Discovery of clinically useful biomarkers and their addition to current predictive models is the new frontier in the era of personalized medicine. In this context, serum ChoE has been investigated in two small retrospective UTUC series [18, 19]. In these studies, low ChoE values were associated with worse RFS, CSS, and/or OS. However, the small patient number and standard statistical analyses adopted limited any definitive conclusion and applicability in clinical practice. We expanded upon these findings by graphically delineating the impact of preoperative serum ChoE as a continuously coded variable on the risk of cancer-specific mortality using Martingale residuals from the underlying Cox proportional hazards model. Such an outcome-based method facilitates the best separation within a cohort between patients at or not at risk of experiencing the outcome of interest. Identifying an optimal cutoff is becoming increasingly central as accurate outcome prediction is paramount to counseling patients and deciding on adjuvant treatment and follow-up strategies in a shared decision-making process.

This is the first study, to our knowledge, to assess the incremental information value of preoperative ChoE levels with a modern statistical approach in UTUC. Previous studies did not investigate the clinical net benefit of their findings on DCA [18, 19]. This is particularly important as conventional multivariable models alone are insufficient to detect a clinically significant biomarker [6]. For instance, our analysis found an increase in the c-indices for all multivariable models predicting survival outcomes after adding ChoE to the reference model. However, despite a significant association of low ChoE levels with survival outcomes in these multivariate analyses, it did not improve the clinical net benefit on DCA.

Hydrolyzing acetylcholine, ChoE intrinsically regulates inflammatory pathways and may interact with immune-mediated tumor development and progression [25]. However, the pathophysiological mechanism associated with decreased serum ChoE levels in different malignancies is not yet understood. Explanations include the interactions between the tumor-host and a tumor-associated chronic inflammatory response as well as tumor-related weight loss or malnutrition [10]. Our study adds to the growing evidence on the role of inflammatory response biomarkers for outcome prediction in urothelial cancer [23, 26, 27]. Therefore, clinical implications of our findings include the consideration to add ChoE to predictive models based on inflammatory response markers to help guide personalized perioperative counseling and decision-making. Furthermore, its role as a predictive marker in the era of immunotherapy could be of further value and, therefore, of research.

Several limitations restrict our findings. First, our study suffers from retrospective data acquisition and analysis despite its multi-centric design. Second, by dichotomizing ChoE using a statistical cutoff, we may have lost some of its predictive capacity and clinically relevant information. In addition, dichotomization may contribute to overestimating the true impact of ChoE on UTUC outcomes. Therefore, we used continuously coded ChoE in the multivariate analyses. Third, we fitted the ChoE cutoff value to our cohort, requiring further external validation of this cutoff in future studies. Fourth, there was no centralized pathology review, there were multiple surgeons, and there was no control for surgeon’s experience. Fifth, our analyses are limited by missing data on intravesical recurrences. Sixth, despite a standardized measurement protocol for ChoE at each institution, measurement inaccuracies may have led to the misclassification of preoperative serum ChoE levels in individual patients.

## Conclusion

Despite its statistically significant association with oncologic outcomes, preoperative serum ChoE has no impact on clinical decision-making. In future studies, ChoE should be investigated as part of the tumor microenvironment and integrated into predictive and prognostic models based on inflammatory response markers, specifically in patients considered for immune checkpoint inhibition therapy.

## Supplementary Information

Below is the link to the electronic supplementary material.

Supplementary file1 (DOCX 20 KB)

Supplementary file2 (DOCX 160 KB)

## Data Availability

Due to the ethical/legal nature of this research, supporting data is not available.
