# Supplementary material for: Clinical value of cholinesterase in patients treated with radical nephroureterectomy for upper urinary tract carcinoma
Source: World J Urol. 2023 Jun 9;41(7):1861–8. doi: 10.1007/s00345-023-04449-1 (PMC10352439; doi:10.1007/s00345-023-04449-1)
Supplement: Supplementary file 1 — Supplementary file1 (DOCX 20 KB) [file 345_2023_4449_MOESM1_ESM.docx]

| **Supplementary Table 1.** Multivariable competing risks regression analysis assessing the association of clinicopathologic features with cancer-specific mortality (CSM) before and after implementation of cholinesterase (ChoE) in 748 patients treated with radical nephroureterectomy for clinically non-metastatic upper tract urothelial cancer. | | | | | | |
| --- | --- | --- | --- | --- | --- | --- |
|  | **CSM** | | | **CSM** | | |
|  | **sHR** | **95% CI** | **p-value** | **sHR** | **95% CI** | **p-value** |
| Stage (ref Ta/Tis) |  |  |  |  |  |  |
| T1 | 1.66 | 0.66-4.19 | 0.3 | 2.03 | 0.86-4.77 | 0.1 |
| T2 | 3.80 | 1.50-9.62 | **0.005** | 2.98 | 1.28-6.94 | **0.011** |
| T3 | 5.79 | 2.35-14.3 | **<0.001** | 5.40 | 2.38-12.3 | **<0.001** |
| T4 | 22.0 | 7.92-61.1 | **<0.001** | 12.4 | 4.27-36.2 | **<0.001** |
| High Grade | 1.68 | 0.97-2.91 | 0.06 | 1.42 | 0.88-2.29 | 0.2 |
| Concomitant CIS | 0.90 | 0.57-1.39 | 0.6 | 0.93 | 0.58-1.51 | 0.8 |
| N stage (ref pN0) |  |  |  |  |  |  |
| pN1 | 1.69 | 0.92-3.11 | 0.09 | 1.65 | 0.84-3.24 | 0.2 |
| pNx | 0.83 | 0.52-1.34 | 0.5 | 0.67 | 0.42-1.06 | 0.08 |
| Adverse pathologic features* | 1.26 | 0.87-1.82 | 0.2 | 1.26 | 0.88-1.79 | 0.2 |
| Perioperative chemotherapy | 1.26 | 0.76-2.08 | 0.4 | 0.74 | 0.42-1.33 | 0.3 |
| ChoE (continuously coded) |  |  |  | 0.59 | 0.53-0.65 | **<0.001** |
| CI = Confidence Interval; sHR = subhazard Ratio | | | | | | |
| * One or multiple features including lymphovascular invasion, tumor necrosis, tumor architecture, and variant histology | | | | | | |
