# Supplementary material for: Clinical value of cholinesterase in patients treated with radical nephroureterectomy for upper urinary tract carcinoma
Source: World J Urol. 2023 Jun 9;41(7):1861–8. doi: 10.1007/s00345-023-04449-1 (PMC10352439; doi:10.1007/s00345-023-04449-1)
Supplement: Supplementary file 2 — Supplementary file2 (DOCX 160 KB) [file 345_2023_4449_MOESM2_ESM.docx]

**Clinical Value of Cholinesterase in Patients Treated with Radical Nephroureterectomy for Upper Urinary Tract Carcinoma**

Markus von Deimling^1,2,*^, David D'Andrea^1,*^, Benjamin Pradere^1,3^, Ekaterina Laukhtina^1,4^, Takafumi Yanagisawa^1,5^, Tatsushi Kawada^1,6^, Muhammad Majdoub^1,7^, Pawel Rajwa^1,8^, Maximilian Pallauf^1,9,10^, Nirmish Singla^9^, Francesco Soria^1,11^, Vitaly Margulis^1,12^, Piotr Chlosta^13^, Pierre I. Karakiewicz^14^, Morgan Roupret^15^, Jeremy Yuen-Chun Teoh^16^, Margit Fisch^2^, Michael Rink^2^, Marco Moschini^17^, Yair Lotan^12^, Shahrokh F. Shariat^1,4,12,18-20^

1. *Department of Urology, Comprehensive Cancer Center, Medical University of Vienna, Vienna, Austria*
2. *Department of Urology, University Medical Center Hamburg-Eppendorf, Hamburg, Germany*
3. *Department of Urology, La Croix Du Sud Hospital, Quint-Fonsegrives, France*
4. *Institute for Urology and Reproductive Health, Sechenov University, Moscow, Russia*
5. *Department of Urology, The Jikei University School of Medicine, Tokyo, Japan*
6. *Department of Urology, Okayama University Graduate School of Medicine, Dentistry and Pharmaceutical Sciences, Okayama, Japan*
7. *Department of Urology, Hillel Yaffe Medical Center, Hadera, Israel*
8. *Department of Urology, Medical University of Silesia, Zabrze, Poland*
9. *Departments of Urology and Oncology, The James Buchanan Brady Urological Institute, Johns Hopkins University School of Medicine, Baltimore, MD, United States*
10. *Department of Urology, University Hospital Salzburg, Paracelsus Medical University, Salzburg, Austria*
11. *Division of Urology, Department of Surgical Sciences, San Giovanni Battista Hospital, University of Studies of Torino, Turin, Italy*
12. *Department of Urology, University of Texas Southwestern Medical Center, Dallas, Texas*
13. *Department of Urology, Jagiellonian University, Cracow, Poland*
14. *Cancer Prognostics and Health Outcomes Unit, Division of Urology, University of Montreal Health Center, Montreal, Canada*
15. *Sorbonne University, GRC 5 Predictive Onco-Uro, AP-HP, Urology, Pitie-Salpetriere Hospital, PARIS, France*
16. *S.H. Ho Urology Centre, Department of Surgery, Prince of Wales Hospital, The Chinese University of Hong Kong, Hong Kong*
17. *Department of Urology, Urological Research Institute, Vita-Salute University, San Raffaele Scientific Institute, Milan, Italy*
18. *Karl Landsteiner Institute of Urology and Andrology, Vienna, Austria*
19. *Department of Urology, Weill Cornell Medical College, New York, New York, USA*
20. *Department of Urology, Second Faculty of Medicine, Charles University, Prague, Czech Republic*

* These authors contributed equally

**Corresponding author:** Prof. Shahrokh F. Shariat

Department of Urology, Comprehensive Cancer Center, Vienna General Hospital, Medical University of Vienna

Währinger Gürtel 18-20, 1090 Vienna, Austria

Email: shahrokh.shariat@meduniwien.ac.at

Tel: + 43 14040026150

Fax: + 43 14040023320

World Journal of Urology

**
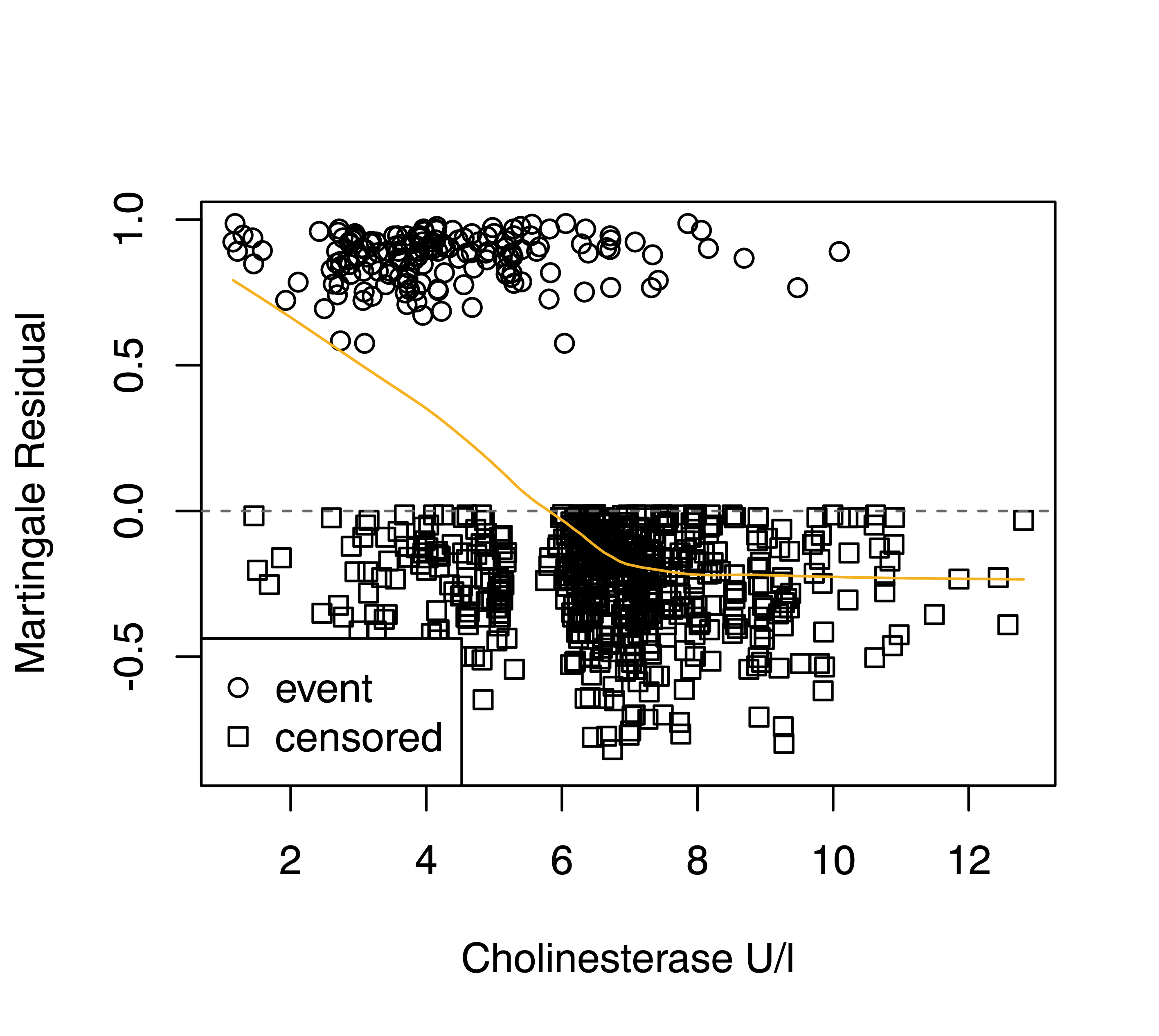
**

**Supplementary Figure 1** – Visual cut-off identification via assessment of the functional form of the association of preoperative serum cholinesterase levels with cancer-specific survival. The plot displays the Martingale residuals from a multivariable Cox model.


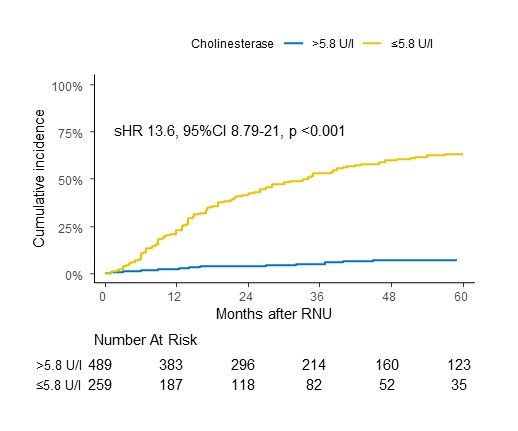


**Supplementary Figure 2** – Univariable competing risks regression for cancer-specific mortality for 748 patients treated with radical nephroureterectomy (RNU) for clinically non-metastatic upper urinary tract urothelial carcinoma stratified by preoperative serum cholinesterase levels.

sHR = subhazard ratio
